# HMGB1 and Kallistatin: Novel Serological Markers for Differentiating Peritonsillar Cellulitis and Abscess

**DOI:** 10.3390/diagnostics15202554

**Published:** 2025-10-10

**Authors:** Kadir Sinasi Bulut, Fatih Gul, Tuba Saadet Deveci Bulut, Burak Celik, Serkan Serifler, Mehmet Ali Babademez

**Affiliations:** 1 Department of Otolaryngology, Head and Neck Surgery, School of Medicine, Ankara Yildirim Beyazit University, Ankara 06800, Turkey; burakcelik70@gmail.com (B.C.); serkanserifler@gmail.com (S.S.); mababademez@gmail.com (M.A.B.); 2 Department of Otolaryngology, Head and Neck Surgery, School of Medicine, Lokman Hekim University, Ankara 06530, Turkey; drfatihgul@gmail.com; 3 Department of Biochemistry, Ankara City Hospital, Ankara 06800, Turkey; tsdbulut@gmail.com

**Keywords:** peritonsillar abscess, cellulitis, HMGB1 protein, serine protease inhibitor, biological markers

## Abstract

**Background/Objectives**: Peritonsillar abscess (PTA) and cellulitis (PTC) often present with similar clinical features, making differentiation challenging despite imaging. This study evaluates the diagnostic performance of serum HMGB1 and kallistatin levels as potential independent biomarkers to distinguish PTA from PTC. **Methods**: In this single-center prospective cohort study, 97 patients aged 18 to 65 years who met the inclusion criteria and presented with peritonsillar infection (39 PTA; 58 PTC) between February and July 2025 were enrolled. Serum levels of HMGB1, kallistatin, and routine inflammatory markers were measured and compared. Univariate and multivariate logistic regression analyses identified independent predictors for distinguishing PTA from PTC. Receiver operating characteristic (ROC) curve analysis assessed the diagnostic accuracy of biomarkers. Decision curve analysis (DCA) was performed to evaluate the clinical net benefit of individual biomarkers and their combinations across a range of threshold probabilities. **Results**: Compared to controls, patients with peritonsillar infection had significantly higher WBC, neutrophil, CRP, procalcitonin, and HMGB1 levels and significantly lower kallistatin levels (all *p* < 0.05). Within the infection group, PTA patients showed significantly higher CRP (*p* = 0.036) and HMGB1 (*p* = 0.003) levels and lower kallistatin (*p* < 0.001) levels compared to PTC patients. In univariate analysis, CRP, HMGB1, and kallistatin were significantly associated with PTA; however, in multivariate analysis, only elevated HMGB1 (OR: 1.21; 95% CI: 1.09–1.35; *p* < 0.001) and reduced kallistatin (OR: 0.395; 95% CI: 0.24–0.648; *p* < 0.001) remained independent predictors. ROC analysis showed that both HMGB1 and kallistatin demonstrated good discriminative ability in distinguishing PTA from PTC. DCA revealed that the three-biomarker combination (kallistatin + HMGB1 + CRP) achieved the highest mean net benefit (0.183) across all threshold probabilities, outperforming individual biomarkers (kallistatin: 0.131, HMGB1: 0.111, CRP: 0.099) and the two-biomarker model (0.176). The combined model maintained superior net benefit across threshold probabilities of 25–75%, indicating optimal clinical utility within this decision range. **Conclusions**: Serum HMGB1 and kallistatin may be effective adjunctive biomarkers for differentiating PTA from PTC.

## 1. Introduction

Peritonsillar abscess (PTA) is one of the most common deep neck infections in otolaryngology practice, with an annual incidence of about 30 cases per 100,000 individuals [1,2]. Peritonsillar cellulitis (PTC) is considered an earlier stage in the same pathological spectrum. PTC is marked by cellulitis but lacks organized abscess formation. The exact relationship and progression between these two conditions remain incompletely understood [2,3,4].

High mobility group box 1 (HMGB1) is a prototypical pro-inflammatory alarmin (DAMP) released during cellular injury and immune activation, whereas kallistatin is a serine proteinase inhibitor (SERPIN) with anti-inflammatory and antioxidant properties. Kallistatin and HMGB1 are both involved in the inflammatory cascade, with kallistatin exerting anti-inflammatory and cytoprotective effects that can counteract HMGB1-mediated pro-inflammatory signaling. Their opposing biological roles suggest a pathophysiological interplay, in which elevated HMGB1 and reduced kallistatin may act synergistically to amplify tissue inflammation and disease severity. Studies show that kallistatin levels drop significantly in severe sepsis and septic shock, with levels inversely related to disease severity. Lower kallistatin is also associated with higher mortality and worse disease progression in community-acquired pneumonia [5,6]. Extracellular HMGB1 is involved in systemic infections like sepsis, and its levels correlate with disease severity [7,8,9].

A recent study reported that 30.4% of computed tomography (CT) scans interpreted as peritonsillar abscess showed no purulence upon intervention, highlighting the potential for diagnostic inaccuracy [10]. Additionally, intraoral ultrasound, although accurate for diagnosing PTA, is often not feasible in patients with severe trismus due to limited mouth opening and patient discomfort. These limitations underscore the need for complementary diagnostic approaches that can improve accuracy, particularly in cases where clinical and radiological findings are inconclusive. Kallistatin and HMGB1, which participate in opposing arms of the inflammatory cascade, may provide additional diagnostic insight through their distinct biological roles. We hypothesized that serum kallistatin and HMGB1 levels could serve as reliable biomarkers to distinguish PTA from PTC, thereby supporting clinical decision-making and potentially reducing unnecessary invasive procedures.

## 2. Materials and Methods

**Figure 1:**
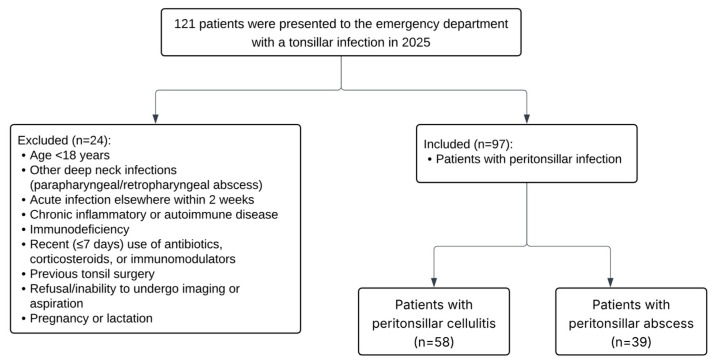
STROBE flow diagram: selection of study participants, reasons for exclusion, and final PTA/PTC distribution.

The research was a prospective cohort study conducted at a single center. This study was conducted on patients who presented to the emergency department of our tertiary training and research hospital and were followed up and treated in the otorhinolaryngology department with a diagnosis of peritonsillar infection between February 2025 and July 2025. A total of 97 patients were included in the study (PTA: *n* = 39; PTC: *n* = 58). The control group consisted of 60 healthy volunteers without chronic comorbidities, recent infections, or use of systemic antibiotics, corticosteroids, or immunomodulatory drugs. All control subjects were confirmed to be free of recent infections or inflammatory conditions based on the absence of clinical symptoms and normal laboratory parameters, including leukocyte, neutrophil, lymphocyte counts, and CRP levels. Demographic data were collected from all participants. Exclusion criteria included patients younger than 18 years; those with other deep neck space infections such as parapharyngeal or retropharyngeal abscess; concurrent acute bacterial or viral infections at another site within the previous two weeks; any chronic inflammatory disease and autoimmune disorders; immunodeficiency; receipt of systemic antibiotics, corticosteroids, or immunomodulatory drugs within seven days prior to blood sampling; prior head and neck surgery involving the tonsillar region; inability or refusal to undergo imaging or aspiration; and pregnancy or lactation (Figure 1).

### 2.1. Definition of PTA and PTC

PTC was diagnosed based on the presence of clinical features consistent with peritonsillar infection (e.g., unilateral sore throat, odynophagia, trismus, and peritonsillar swelling) in the absence of fluctuance on physical examination, no evidence of purulent collection on contrast-enhanced computed tomography, and failure to obtain pus on fine-needle aspiration. In cases with initial diagnostic uncertainty, a rapid clinical improvement within 24–48 h of antibiotic therapy without surgical drainage further supported the diagnosis of PTC. PTA presents with a clinical picture similar to PTC but is characterized by the presence of a localized purulent collection in the peritonsillar space, confirmed by fluctuance on examination, imaging evidence on contrast-enhanced computed tomography, and aspiration of pus.

### 2.2. Measurement of Serum Kallistatin and HMGB1 Levels

Venous blood samples were obtained from all participants in the emergency department at the time of diagnosis, prior to the initiation of antibiotic or anti-inflammatory treatment. Samples were collected into serum separator tubes and allowed to clot at room temperature for 30 min, then centrifuged at 900× *g* for 10 min. The separated serum was aliquoted and stored at −80 °C until analysis. Serum concentrations of kallistatin and HMGB1 were determined using commercially available ELISA kits (Bioassay Technology Laboratory, Shanghai, China; catalog No. E1635Hu for HMGB1 and E3392Hu for kallistatin), according to the manufacturer’s instructions. Absorbance was measured at 450 nm with a microplate reader, and concentrations were calculated from standard curves generated using appropriate calibrators. The limits of detection were 0.022 ng/mL for kallistatin and 0.24 ng/mL for HMGB1. The intra- and inter-assay coefficients of variation for both assays were <8% and <10%, respectively. Routine hematological and biochemical parameters, including complete blood count, C-reactive protein, procalcitonin, and albumin, were assessed using standard automated methods in the hospital’s central laboratory.

### 2.3. Statistical Analyses

Statistical analyses were performed using IBM SPSS Statistics for MacOS, Version 30.0 (IBM Corp., Armonk, NY, USA). Sample size calculation was performed a priori using G*Power software (version 3.1.2), based on previously published variance estimates of kallistatin levels in peritonsillar infections [11]. Assuming a two-sided α of 0.05 and aiming for 95% power to detect a clinically meaningful difference, the required minimum sample size was estimated as 24 patients per group (total *n* = 48; noncentrality parameter δ = 3.40; critical t = 1.68; df = 46; actual power = 0.956). Our study enrolled 39 patients with PTA and 58 patients with PTC, thus exceeding the minimum requirement and ensuring adequate statistical power. Continuous variables were expressed as the mean ± standard deviation (SD) and compared between groups using the independent samples *t*-test. Receiver operating characteristic (ROC) curve analysis was conducted to evaluate the diagnostic performance of HMGB1 and kallistatin in distinguishing PTA from PTC, and the area under the curve (AUC) with 95% confidence intervals (CI) was calculated; optimal cut-off values were determined using the Youden index. Variables with clinical relevance or *p* < 0.05 in comparative analyses were entered into univariable logistic regression, and those with predictive effect were subsequently evaluated in a multivariable model. For the logistic regression analysis, we achieved an events-per-variable (EPV) ratio of 13 (39 PTA cases divided by 3 predictor variables: CRP, HMGB1, and kallistatin). This exceeds the widely accepted minimum threshold of 10 EPV, recommended by Peduzzi et al. (1996) and Vittinghoff & McCulloch (2007), for stable regression estimates and a reduced overfitting risk [12,13]. In addition, sensitivity, specificity, and their 95% confidence intervals at optimal cut-off values were calculated, along with positive and negative predictive values at different prevalence scenarios. Additionally, decision curve analysis (DCA) was performed using R software (version 4.5.1; R Foundation for Statistical Computing, Vienna, Austria) to evaluate the clinical net benefit of individual biomarkers and their combinations across a range of threshold probabilities. A *p*-value < 0.05 was considered statistically significant.

## 3. Results

A total of 157 subjects were included in the study: 97 in the infection cohort (39 with peritonsillar abscess and 58 with peritonsillar cellulitis; 50 males and 47 females) and 60 in the control cohort (34 males and 26 females). All numerical variables were normally distributed (Shapiro–Wilk *p* > 0.05 across groups). There was no statistically significant difference in mean age between the infection group (37.74 ± 12.47 years) and the control group (40.93 ± 12.50 years; *p* = 0.564). Compared with controls, patients with peritonsillar infection showed significantly elevated White Blood Cell (WBC) counts (16.08 ± 3.02 vs. 6.84 ± 1.47 × 10^3^/μL; *p* < 0.001), neutrophil counts (12.71 ± 3.06 vs. 3.97 ± 1.05 × 10^3^/μL; *p* < 0.001), CRP levels (100.23 ± 85.24 vs. 14.60 ± 8.03 mg/L; *p* < 0.001), procalcitonin levels (0.15 ± 0.20 vs. 0.09 ± 0.06 ng/mL; *p* = 0.038), and HMGB1 concentrations (21.02 ± 7.61 vs. 16.12 ± 4.10 ng/mL; *p* < 0.001). In contrast, kallistatin concentrations were significantly lower in the infection group than in controls (3.65 ± 2.02 vs. 5.95 ± 2.50 ng/mL; *p* < 0.001). No significant between-group differences were observed for lymphocyte counts (2.03 ± 0.98 vs. 2.18 ± 0.51 × 10^3^/μL; *p* = 0.262) or albumin levels (45.42 ± 3.66 vs. 45.20 ± 3.23 g/L; *p* = 0.713) (Table 1).

Within the infection cohort, patients were divided into two subgroups: PTA (*n* = 39) and PTC (*n* = 58). CRP levels were higher in the PTA subgroup (122.30 ± 79.08 mg/L) than in the PTC subgroup (85.39 ± 86.66 mg/L; *p* = 0.036). WBC, neutrophil, lymphocyte, and procalcitonin values were similar in both groups (*p* > 0.005). HMGB1 concentrations were greater in PTA (23.82 ± 8.40 ng/mL) than in PTC (19.13 ± 6.44 ng/mL; *p* = 0.003). Kallistatin levels were correspondingly lower in the PTA (2.76 ± 1.57 vs. 4.25 ± 2.08 ng/mL; *p* < 0.001) (Table 2).

**Figure 2:**
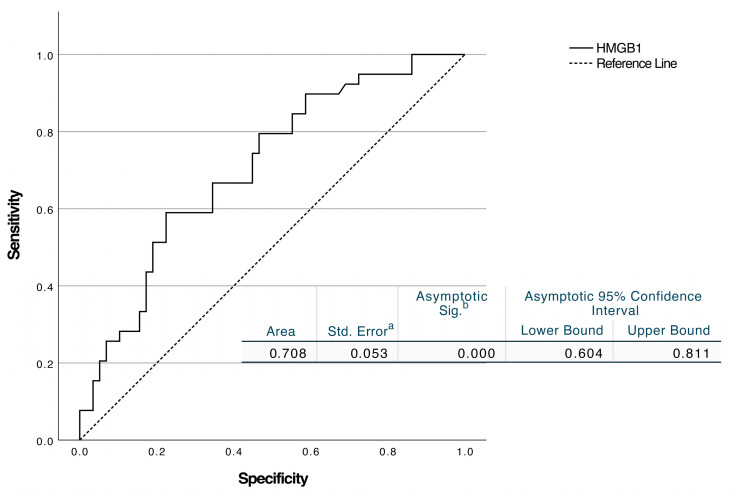
ROC analysis of HMGB1 levels to discriminate peritonsillar abscess from peritonsillar cellulitis. (a) Standard error computed using the DeLong method. (b) Null hypothesis: true area = 0.5.

**Figure 3:**
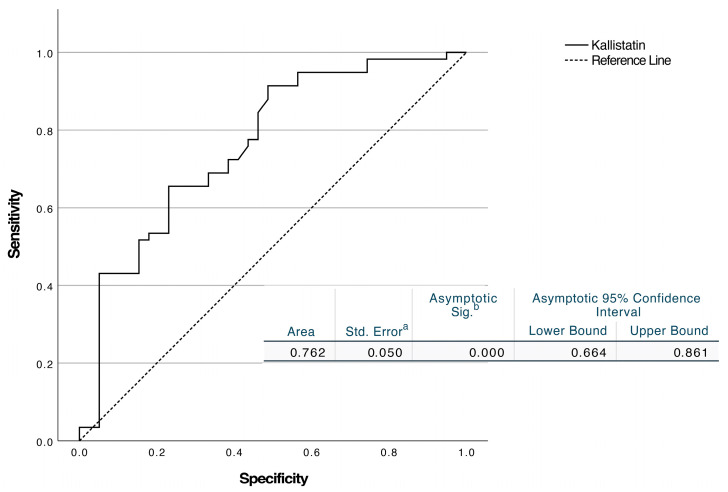
ROC analysis of kallistatin levels to discriminate peritonsillar abscess from peritonsillar cellulitis. (a) Standard error computed using the DeLong method. (b) Null hypothesis: true area = 0.5.

ROC curve analysis revealed that HMGB1 had an AUC of 0.708 (95% CI, 0.604–0.811; *p* < 0.001). A cut-off of ≥18.79 ng/mL resulted in 66.7% sensitivity (95% CI: 51–79.4%) and 65.5% specificity (95% CI: 52.7–76.4%) (Figure 2). Kallistatin had an AUC of 0.762 (95% CI 0.664–0.861; *p* < 0.001). The threshold of ≤2.61 ng/mL showed 72.4% sensitivity (95% CI: 59.8–82.2%) and 61.5% specificity (95% CI: 45.9–75.1%) (Figure 3).

We performed univariate and multivariate logistic regression analyses to identify factors associated with PTA and to determine which variables were independent predictors after adjusting for potential confounders. In univariate logistic regression analysis, higher CRP (OR: 1.005; 95% CI: 1.00–1.01; *p* = 0.042), elevated HMGB1 (OR: 1.092; 95% CI: 1.026–1.162; *p* = 0.006), and lower kallistatin (OR: 0.587; 95% CI: 0.423–0.815; *p* = 0.001) levels were significantly associated with the presence of PTA. Variables that were statistically significant in the univariate analysis were included in the multivariate analysis. CRP was not a significant predictor (B = 0.004; *p* = 0.162; Odds Ratio (OR) 1.004; 95% CI 0.998–1.010). In contrast, HMGB1 (B = 0.194; *p* < 0.001; OR 1.21; 95% CI 1.09–1.35) and kallistatin (B = −0.930; *p* < 0.001; OR 0.395; 95% CI 0.24–0.648) were independent determinants of abscess formation (Table 3). In the univariate analysis, each 1 ng/mL increase in HMGB1 was linked to a 9% higher odds of peritonsillar abscess. Conversely, higher kallistatin levels were protective, corresponding to an approximate 41% reduction in odds. In the multivariate model, HMGB1 persisted as an independent risk factor, with each 1 ng/mL increase associated with a 21% higher odds, while kallistatin retained its protective effect, corresponding to an approximate 60% reduction in odds.

At the observed prevalence in our study population (40.2%), HMGB1 and kallistatin demonstrated similar predictive values, with PPVs of 56.5% and 55.8%, and NPVs of 74.5% and 76.8%, respectively. Given that PTA prevalence in clinical practice may vary, we calculated PPV and NPV at different prevalence scenarios (Table 4). At a lower prevalence of 20%, both biomarkers showed moderate PPVs (HMGB1: 32.6%, kallistatin: 31.5%) but high NPVs (HMGB1: 88.7%, kallistatin: 89.9%), indicating that they perform better for ruling out PTA than ruling in PTA in low-prevalence settings.

Bacterial culture results are summarized in Table 5. Among patients with PTA, *Streptococcus pyogenes* (6/39) was the most frequently isolated pathogen, followed by polymicrobial growth (4/39). In the PTC group, the most common findings were no bacterial growth (22/58) and polymicrobial cultures (4/58). In both groups, the rate of isolating a single pathogen was generally low. Notably, cultures were not performed in 7 cases of PTA and 25 cases of PTC, meaning bacterial data were unavailable for these patients.

**Figure 4:**
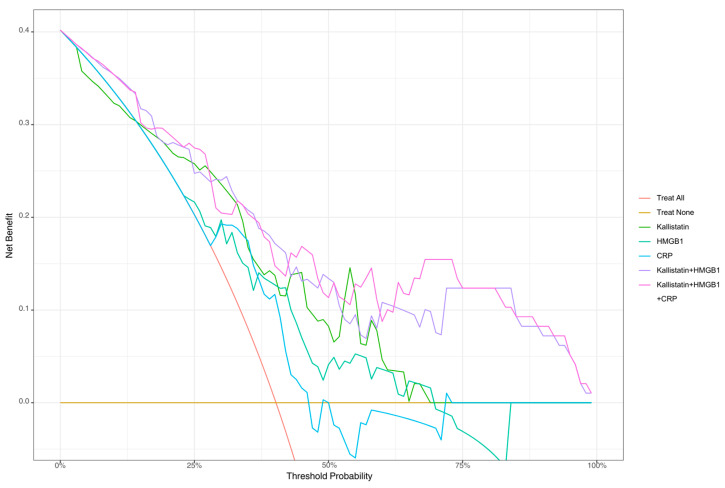
Clinical Utility of Kallistatin, HMGB-1, and CRP Biomarkers: Decision Curve Analysis.

Decision curve analysis demonstrated that all models achieved a maximum net benefit of 0.402 at a threshold probability 0% (treat all strategy). However, the three-biomarker combination model showed the highest mean net benefit (0.183) across all threshold probabilities (Table 6). The two-biomarker combination achieved a mean net benefit of 0.176, while individual biomarkers showed lower values (kallistatin: 0.131, HMGB-1: 0.111, CRP: 0.099). At clinically relevant threshold probabilities, the three-biomarker combination maintained the highest net benefit at a 25% threshold (0.275), while demonstrating sustained clinical utility at 50% (0.113) and 75% (0.124) thresholds. In contrast, individual biomarkers showed diminishing or negative net benefit at higher thresholds, with HMGB-1 showing negative net benefit (−0.031) at 75% threshold probability (Figure 4).

## 4. Discussion

PTA and PTC represent two stages within the spectrum of peritonsillar infection and often present with overlapping clinical features, making differentiation challenging in the acute setting. CT can aid in diagnosis; however, false-positive results and unsuccessful fine-needle aspirations underscore the need for complementary diagnostic tools [10,14,15]. In this prospective cohort investigation, we have evaluated the biomarker roles of HMGB1 and kallistatin in differentiating PTA from PTC. We found that serum HMGB1 levels were significantly higher and serum kallistatin levels were significantly lower in PTA compared to PTC. Although CRP, HMGB1, and kallistatin were all significantly associated with PTA in univariate analysis, only HMGB1 and kallistatin remained independently associated in the multivariate model, indicating superior diagnostic associations after adjustment for confounders. These findings highlight the potential utility of HMGB1 and kallistatin support the potential utility of HMGB1 and kallistatin as adjunctive biomarkers, particularly in cases where clinical and radiological findings are inconclusive and may help reduce reliance on invasive diagnostic procedures.

An abscess is a localized focus of inflammation that typically develops as a result of bacterial infection and is characterized by intense neutrophil infiltration, cell death, and accumulation of purulent fluid. HMGB1, a nuclear damage-associated molecular pattern protein, is released from damaged and necrotic cells at the site of infection, acting as an alarmin; it triggers inflammatory cascades via Toll-like receptor and receptor for advanced glycation end-products pathways, stimulates the release of pro-inflammatory cytokines such as TNF-α, IL-1β, and IL-6 from macrophages, enhances neutrophil recruitment, and sustains local inflammation through the formation of neutrophil extracellular traps [9,16,17,18]. As a central mediator in the pathogenesis of sepsis, elevated HMGB1 levels reflect increased tissue damage [19,20]. In contrast, kallistatin, a serine protease inhibitor with anti-inflammatory, antioxidant, and anti-apoptotic properties, antagonizes tissue kallikrein activity, reduces circulating HMGB1 levels, prevents its binding to receptors, and suppresses TNF-α-induced NF-κB activation to limit inflammatory amplification [21,22,23]. Studies have shown that kallistatin levels decrease in sepsis and severe infections and are inversely correlated with disease severity [6]. Therefore, low kallistatin levels during the development of abscess may indicate depletion of endogenous anti-inflammatory reserves, allowing uncontrolled inflammation to progress and resulting in greater damage to surrounding tissues [24].

In current clinical practice, clinicians differentiate PTA from PTC primarily through physical examination findings and use contrast-enhanced CT in equivocal cases. However, CT imaging may present clear drawbacks, including radiation exposure, cost, and limited accessibility [3,14,15]. Relying on serum HMGB1 and kallistatin measurements may assist in distinguishing PTA from PTC. This approach could spare patients unnecessary invasive drainage procedures and the associated discomfort or complications. In addition, monitoring PTC with these biomarkers could enable early detection of abscess formation, allowing timely intervention and thereby preventing potential complications related to PTA. Blood-based biomarkers offer the ability to overcome these limitations by providing rapid, point-of-care results that expedite clinical decision-making in emergency settings.

Few biomarker studies exist in peritonsillar infections, limiting direct comparison. Procalcitonin has shown utility for identifying bacterial involvement in deep neck infections [1,22,23,25,26]. While our study found it elevated in the infection cohort versus controls, it did not differentiate PTA from PTC. This suggests its value in detecting infection, not severity. CRP did not retain significance in our multivariable logistic regression model, likely reflecting its role as a nonspecific acute-phase reactant elevated in both abscess and cellulitis cases. In contrast, HMGB1 (OR 1.21; 95% CI 1.09–1.35) and kallistatin (OR 0.395; 95% CI 0.24–0.648) were independent predictors, suggesting these markers more specifically distinguish PTA from PTC. ROC analysis indicates that both biomarkers possess good discriminative power (AUC > 0.7 for all). Kallistatin demonstrated superior performance with an AUC of 0.762, while HMGB1’s AUC was 0.708. The optimal cut-off for kallistatin (≤2.61 ng/mL) yielded 72.4% sensitivity and 61.5% specificity, and the HMGB1 threshold (≥18.79 ng/mL) provided 66.7% sensitivity and 65.5% specificity. Although these metrics alone may be insufficient for definitive clinical decision-making, combining them with clinical findings could enhance diagnostic accuracy. Notably, assessing HMGB1 and kallistatin together may provide the highest discriminative performance compared with using either biomarker alone. This approach likely reflects the combined pathophysiological contributions of elevated pro-inflammatory activity (HMGB1) and decreased anti-inflammatory capacity (kallistatin) in PTA, enhancing diagnostic performance by capturing a broader spectrum of inflammatory-cascade dynamics. Incorporating currently unmeasured metabolites may be anticipated to further elevate sensitivity to substantially higher levels. This multi-biomarker strategy capitalizes on the complementary functions of pro-inflammatory HMGB1 and the protective effects of kallistatin, thereby maximizing its potential for clinical application.

Our decision curve analysis findings demonstrate that the multi-biomarker approach provides tangible clinical benefit beyond statistical significance in real-world decision-making. The mean net benefit of 0.183 for the three-biomarker combination (kallistatin + HMGB-1 + CRP) indicates that this model would improve clinical decision-making in approximately 18 per 100 patients. This value is consistent with the 0.15–0.25 range typically observed for established biomarker panels in routine clinical use, such as troponin for myocardial infarction or D-dimer for pulmonary embolism [27,28]. Higher net benefit values (>0.40–0.50) are predominantly achieved with imaging modalities or invasive diagnostic procedures; however, these methods carry higher costs, risks, and resource requirements.

The loss of CRP’s significance in the multivariate model is consistent with the DCA results. CRP’s lowest mean net benefit when used alone (0.099) and its inability to provide clinical utility at high threshold values reflect its nature as a nonspecific acute-phase reactant that can be elevated in both abscess and cellulitis. In contrast, HMGB1 and kallistatin’s persistence as independent predictors in multivariate analysis and their higher mean net benefits in DCA (0.111 and 0.131, respectively) support that these markers are more specific for differentiating PTA from PTC.

One of the most significant findings from the DCA is the increase in net benefit from the two-biomarker model to the three-biomarker model (from 0.176 to 0.183). This increase demonstrates that adding the third biomarker (CRP), despite its limited standalone performance, contributes to the combined model. This finding supports the clinical value of developing multi-biomarker panels and indicates that biomarkers reflecting different pathophysiological pathways create a synergistic effect when used together. The anti-inflammatory properties of kallistatin, the pro-inflammatory activity of HMGB1, and CRP’s reflection of the general inflammatory response demonstrate that these three markers provide complementary information.

Our threshold probability analysis provides important insights for clinical application. All models’ superiority over “treat all” and “treat none” strategies across a broad threshold range (5–85%) demonstrates that the biomarker-based approach provides genuine decision-making advantages to clinicians. The achievement of maximum net benefit, particularly in the 25–75% threshold range, indicates that the model is most reliable at these decision thresholds and is most appropriate for use within this range in clinical practice. Interestingly, HMGB1’s demonstration of negative net benefit (−0.031) at high threshold values (75%) shows that this marker remains limited when used alone in situations requiring high specificity, but this limitation is overcome in combined models.

Based on these findings, a simple biomarker-based clinical workflow can be proposed for patients with suspected peritonsillar infection: (i) initially evaluate HMGB1 and kallistatin levels; (ii) if both results are non-significant (HMGB1 < 18.79 ng/mL and kallistatin > 2.61 ng/mL), consider conservative treatment with close clinical follow-up, recognizing a low probability of abscess; (iii) if both biomarkers show significant threshold values (HMGB1 ≥ 18.79 ng/mL and kallistatin ≤ 2.61 ng/mL), prioritize imaging or aspiration/drainage due to high abscess probability; and (iv) in cases showing discordant or intermediate values (e.g., one biomarker positive and the other negative), integrate CRP levels and clinical features (trismus severity and physical examination findings) to reduce diagnostic uncertainty. This algorithm could reduce unnecessary invasive procedures while ensuring early intervention in critical cases, particularly in emergency department settings where imaging is not immediately available or in resource-limited clinics.

One of the strengths of our study is the evaluation of the true clinical utility of models using decision curve analysis, beyond ROC analysis and other traditional performance metrics. While traditional metrics (sensitivity, specificity, AUC) assess diagnostic accuracy, DCA provides practical information to clinicians about which clinical scenarios and at what risk tolerance levels the model will be most beneficial by demonstrating the net benefit of the model at different decision thresholds. This approach provides more meaningful and applicable data for the clinical implementation of biomarkers.

For a rapid, non-invasive test that can be performed on routine blood samples, the observed net benefit value of 0.183 provides significant incremental value. This is clinically meaningful, particularly in emergency department settings or resource-limited environments where advanced imaging modalities are not immediately available. The 85% improvement over individual biomarkers (from the mean net benefit of 0.10–0.13 to 0.183) further emphasizes the clinical advantage of our multi-biomarker approach. When prospectively validated and combined with routine clinical assessment, HMGB1 and kallistatin measurements could enable risk-based triage of patients with suspected peritonsillar infection, reduce diagnostic uncertainty, and help avoid unnecessary invasive procedures (e.g., needle aspiration, cross-sectional imaging, or operative drainage) in low-risk patients.

Building upon these considerations, the strengths of our study include its prospective design, a homogeneous patient population, and standardized laboratory methodologies. Furthermore, diagnoses were objectively confirmed by contrast-enhanced CT scans, and all blood samples were drawn at the time of initial presentation, allowing for assessment independent of disease progression. If prospectively validated and combined with routine clinical assessment, HMGB1 and kallistatin measurements could enable risk-based triage of suspected peritonsillar infection, reducing diagnostic uncertainty and helping to avoid unnecessary invasive procedures (e.g., needle aspiration, cross-sectional imaging, or operative drainage) in low-risk patients.

Our study demonstrates several limitations. First, its single-center nature restricts generalizability. Second, while our sample size is adequate for detecting biomarker differences, larger multicenter cohorts are needed to validate optimal cut-off values and evaluate the prognostic utility for clinical outcomes, such as length of hospital stay and complication rates. Third, the absence of follow-up data prevents analysis of biomarker dynamics in relation to treatment response and long-term prognosis. Fourth, patients with CT findings suggestive of abscess but negative aspiration/drainage were excluded from the study. While this approach minimized the risk of misclassification, it may have introduced selection bias and limited applicability to the full spectrum of patients encountered in clinical practice. Additionally, the small number of culture-positive cases within individual bacterial subgroups limited our ability to evaluate pathogen-specific associations with biomarker levels. Finally, we did not conduct a cost-effectiveness analysis of these biomarkers. Moreover, the fact that sensitivity and specificity did not reach very high levels may limit the perceived diagnostic conclusiveness of the findings. However, the increase observed in the ratio-based analysis could be considered a promising result, as it suggests that incorporating additional parameters from the cascade may further enhance diagnostic accuracy.

Future studies should include larger, multicenter cohorts to confirm and extend our findings. Investigations correlating biomarker levels with therapeutic responses and patient outcomes are warranted, as are evaluations of their diagnostic value in other deep neck infections.

## 5. Conclusions

Elevated HMGB1 and reduced kallistatin levels are independent markers for distinguishing PTA from PTC. Decision curve analysis confirms that the three-biomarker combination (kallistatin + HMGB-1 + CRP) provides clinically meaningful net benefit (mean 0.183), outperforming individual biomarkers and maintaining utility across threshold probabilities of 25–75%. Incorporating these biomarkers into clinical assessment may improve diagnostic accuracy, reduce unnecessary imaging and invasive procedures, and enable early detection of abscess formation during cellulitis follow-up, thereby preventing complications. A tiered clinical workflow utilizing HMGB1 and kallistatin for initial risk stratification, supplemented by CRP in equivocal cases, could optimize resource utilization in emergency and resource-limited settings. Larger multicenter studies are needed to validate these findings and evaluate their impact on clinical outcomes.

## Figures and Tables

**Table 1:** Comparison of demographic and laboratory parameters between the peritonsillar infection group and the control group.

	Peritonsillar Infection Group (*n* = 97)	Control Group (*n* = 60)	
	Mean ± SD	Mean ± SD	*p*-Value
Age, y	37.74 ± 12.47	40.93 ± 12.50	0.564
WBC, 10^3^/μL	16.08 ± 3.02	6.84 ± 1.47	<0.001
Neutrophil, 10^3^/μL	12.71 ± 3.06	3.97 ± 1.05	<0.001
Lymphocyte, 10^3^/μL	2.03 ± 0.98	2.18 ± 0.51	0.262
CRP, mg/L	100.23 ± 85.24	14.60 ± 8.03	<0.001
Procalcitonin, ng/mL	0.15 ± 0.20	0.09 ± 0.06	0.038
Albumin, g/L	45.42 ± 3.66	45.20 ± 3.23	0.713
HMGB1, ng/mL	21.02 ± 7.61	16.12 ± 4.10	<0.001
Kallistatin, ng/mL	3.65 ± 2.02	5.95 ± 2.50	<0.001

Abbreviations: WBC, White Blood Cell; CRP, C-reactive protein; HMGB1, High mobility group box 1.

**Table 2:** Comparative Laboratory Assessment of Peritonsillar Abscess and Cellulitis.

	Peritonsillar Abscess Group (*n* = 39)	Peritonsillar Cellulitis Group (*n* = 58)	
	Mean ± SD	Mean ± SD	*p*-Value
WBC, 10^3^/μL	16.52 ± 4.05	15.79 ± 2.06	0.243
Neutrophil, 10^3^/μL	13.14 ± 4.14	12.43 ± 2.03	0.263
Lymphocyte, 10^3^/μL	1.92 ± 0.62	2.10 ± 1.16	0.387
CRP, mg/L	122.30 ± 79.08	85.39 ± 86.66	0.036
Procalcitonin, ng/mL	0.16 ± 0.27	0.14 ± 0.15	0.557
HMGB1, ng/mL	23.82 ± 8.40	19.13 ± 6.44	0.003
Kallistatin, ng/mL	2.76 ± 1.57	4.25 ± 2.08	<0.001

Abbreviations: WBC, White Blood Cell; CRP, C-reactive protein; HMGB1, High mobility group box 1.

**Table 3:** Univariate Analysis of Individual Variable Associations and Multivariate Analysis of Independent Variable Effects Controlling for Confounders.

	Univariate Analysis					Multivariate Analysis				
				95% C.I for EXP(B)					95% C.I for EXP(B)	
	B	*p*	Exp(B)	Lower	Upper	B	*p*	Exp(B)	Lower	Upper
CRP	0.005	0.042	1.005	1.00	1.01	0.004	0.162	1.004	0.998	1.01
HMGB1	0.088	0.006	1.092	1.026	1.162	0.194	<0.001	1.21	1.09	1.35
Kallistatin	−0.533	0.001	0.587	0.423	0.815	−0.930	<0.001	0.395	0.24	0.648

Abbreviations: CRP, C-reactive protein; HMGB1, High mobility group box 1.

**Table 4:** Predictive Values of Biomarkers at Realistic Disease Prevalences.

Biomarker	Prevalences (%)	PPV (%)	NPV (%)
HMGB1	20	32.6	88.7
	30	45.3	82.1
	40.2 *	56.5	74.5
Kallistatin	20	31.5	89.9
	30	44.1	84.0
	40.2 *	55.8	76.8

Abbreviations: PPV, Positive Predictive Value; NPV, Negative Predictive Value. * Study Population Prevalence.

**Table 5:** Bacterial Culture Results in Patients with PTA and PTC.

	PTA (*n* = 39)	PTC (*n* = 58)
Polymicrobial	4	4
*Streptococcus pyogenes*	6	4
*Streptococcus anginosus*	2	1
*Streptococcus mitis*	2	1
*Streptococcus constellatus*	1	0
*Streptococcus salivarius*	1	0
*Staphylococcus* spp.	2	0
*Haemophilus influenzae*	0	1
No bacterial growth	14	22
Culture not performed	7	25

Abbreviations: PTA (peritonsillar abscess), PTC (peritonsillar cellulitis).

**Table 6:** Decision Curve Analysis Performance Metrics.

Model	Mean Net Benefit	Net Benefit 25%	Net Benefit 50%	Net Benefit 75%
Kallistatin + HMGB-1 + CRP	0.183	0.275	0.113	0.124
Kallistatin + HMGB-1	0.176	0.247	0.134	0.124
Kallistatin	0.131	0.258	0.083	0.000
HMGB-1	0.111	0.216	0.041	−0.031
CRP	0.099	0.203	0.000	0.000

Abbreviations: CRP, C-reactive protein; HMGB-1, High mobility group box 1.

## Data Availability

The data presented in this study are available from the corresponding author upon request. The data are not publicly available due to privacy and ethical restrictions.

## Abbreviations

The following abbreviations are used in this manuscript:

PTA	Peritonsillar Abscess
PTC	Peritonsillar Cellulitis
HMGB1	High Mobility Group Box 1
CRP	C-Reactive Protein
CT	Computed Tomography
ELISA	Enzyme-Linked Immunosorbent Assay
AUC	Area Under the Curve
ROC	Receiver Operating Characteristic
CI	Confidence Interval
OR	Odds Ratio
WBC	White Blood Cell
DCA	Decision Curve Analysis
EPV	Events per Value
